# Exclusion of *PINK1 *as candidate gene for the late-onset form of Parkinson's disease in two European populations

**DOI:** 10.1186/1477-5751-4-10

**Published:** 2005-12-14

**Authors:** Anna Melissa Schlitter, Martin Kurz, Jan P Larsen, Dirk Woitalla, Thomas Mueller, Joerg T Epplen, Gabriele Dekomien

**Affiliations:** 1 Department of Human Genetics, Ruhr-University Bochum, Germany; 2 Department of Neurology, Heinrich-Heine-University Düsseldorf, Germany; 3 Department of Neurology, Stavanger University Hospital, Norway; 4 Department of Neurology, St. Josef-Hospital, Ruhr-University Bochum, Germany

## Abstract

**Background:**

Parkinson's disease (PD) is the second most common neurodegenerative disorder. Recently, mutations in the *PINK1 *(PARK6) gene were shown to rarely cause autosomal-recessively transmitted, early-onset parkinsonism. In order to evaluate whether *PINK1 *contributes to the risk of common late-onset PD we analysed *PINK1 *sequence variations. A German (85 patients) and a Norwegian cohort (90 patients) suffering from late-onset PD were screened for mutations and single nucleotide polymorphisms (SNPs) in the *PINK1 *gene. Both cohorts consist of well-characterized patients presenting a positive family history of PD in ~17%. Investigations were performed by single strand conformation polymorphism (SSCP), denaturating high performance liquid chromatography (DHPLC) and sequencing analyses. SNP frequencies were compared by the χ^2 ^test

**Results:**

Several common SNPs were identified in our cohorts, including a recently identified coding variant (Q115L) in exon 1. Genotyping of the Q115L variation did not reveal significant frequency differences between patients and controls. Pathogenic mutations in the *PINK1 *gene were not identified, neither in the German nor in the Norwegian cohort.

**Conclusion:**

Sequence variation in the *PINK1 *gene appears to play a marginal quantitative role in the pathogenesis of the late-onset form of PD, in German and Norwegian cohorts, if at all.

## Background

PD is the second most common neurodegenerative disorder after Alzheimer disease affecting more than 1% of the population by the age of 65 years. Mutations in the *alpha-synuclein *(PARK1), *Parkin *(PARK2) and *DJ-1 *(PARK7) gene cause fairly rare familial forms of PD characterized by an early age of onset. Mutations in the recently identified *LRRK2 *(PARK8) gene, especially the common mutation G2019S, occur more frequently in patients suffering from early as well as late-onset PD [1,2]. Recently, mutations in the *PINK1 *(PARK6) gene were shown to cause autosomal recessively transmitted early-onset parkinsonism [3,4]. The PINK1 (PTEN-induced kinase 1) gene encodes a putative protein kinase. The protein is targeted to mitochondria and shows a serine-threonine kinase domain with homology to kinases of the Ca^2+^/calmodulin family[3]. It appears to exert protective effects against cellular stress within mitochondria[3]. These findings link mitochondria directly to the pathogenesis of PD [3,5]. An additional link between mitochondrial dysfunction and PD is obvious via the identification of disease causing mutations in the *Omi/HtrA2 *gene [6]. The hypothesis of mitochondrial impairment was further emphasized by postmortem studies of PD brains [7] and observation of PD syndromes after intoxication with mitochondrial complex I inhibitors, such as MPTP (1-methyl-4-phenyl-1,2,3,6-tetrahydropyridine) and rotenone [8]. Mutations in the *PINK1 *gene are the second most common cause of autosomal-recessively inherited early-onset PD after mutations in the *Parkin *gene. On the other hand, strong evidence was reported for a possible role of *Parkin *gene variations in the late-onset form of PD (age of onset >45 years): *Parkin *mutations appear to contribute to the common late-onset form and mutations, especially in exon 7 in heterozygous state, may play a role as susceptibility alleles for sporadic PD[9,10]. The question arises as to whether the *PINK1 *gene is also a candidate gene for late-onset forms of Parkinson's disease, similar to the suggested role of the *Parkin *gene.

Here we report of a population-based analysis of sequence variations within the *PINK1 *gene. The German cohort includes 85 patients suffering from late-onset form of PD and represents a modern urban population with a genetic heterogeneity. In contrast, the Norwegian cohort represents a more homogeneous population [11] and includes 90 patients suffering from late-onset form of PD.

## Results

All 8 coding exons of the *PINK1 *gene were screened for sequence variation using SSCP and sequencing analyses. Several SNPs were identified in our cohorts (L63L, Q115L, Ivs4-5A>G het, Ivs6+43C>T het, N521T, c.1783A>T). Allelic frequencies of several SNPs differed significantly between the two European cohorts confirming homogeneity of the Norwegian cohort (Table 1). Patients and controls were genotyped for the recently identified variation Q115L [12] using DHPLC analysis (Table 2). The observed frequencies did not differ significantly between patients and controls, neither in the German (p = 0.27) nor in the Norwegian cohort (p = 0.8). This screening did not reveal any disease-relevant mutation in our cohorts.

**Table 1:** Allelic frequencies of identified SNPs in Norwegian (NW) and German (G) patient cohorts

Exon	Sequence variation	Allelic frequencies		p-value *distribution is significant
		NW	G	
1	Q115L	0.072	0.035	0.13
1	L63L	0.083	0.218	0.0004 *
5	Ivs4-5A>G	0.017	0.091	0.002 *
6	Ivs6+43C>T	0.041	0.102	0.029 *
8	N521T	0.128	0.188	0.13
8	c.1783A>T	0.201	0.2	0.98

**Table 2:** Genotyping of the Q115L variation

	Norwegian cohort			German cohort		
	Patients (n = 90)	Controls (n = 136)	p	Patients (n = 85)	Controls (n = 210)	p
Q115 (Wildtype)	80	118	0.8	79	190	0.27
Q115L	7	18		6	16	
L115	3	0		0	4	

## Discussion

As recently shown, mutations in the *PINK1 *gene rarely cause autosomal-recessively transmitted PD [3]. Besides an early age of onset, the observed clinical symptoms in PD caused by *PINK1 *are similar to symptoms in idiopathic PD.

In this population-based study, we investigated whether sequence variations in the *PINK1 *gene play a role in the late-onset form of PD. A recently described variation (Q115L) of the *PINK1 *gene [12] was identified in the German and the Norwegian cohorts. We calculated allele frequencies in cases and controls and show here that the Q115L variant was not associated with late-onset PD in our study. These findings correspond to previously published data of no leading association of other coding SNPs within the *PINK1 *gene and PD [13]. In addition, several common SNPs were identified. We did not find any pathogenic mutation of the *PINK1 *in our cohorts composed of patients suffering from late-onset form of PD. The risk to miss potential mutations was minimized by a well established and optimized SSCP analyses.

The most likely reason to explain the absence of mutations in our cohorts is the lack of influence of the *PINK1 *gene in the pathogenesis of late-onset PD. Individual tagging SNPs and tag-defined haplotypes in 500 PD patients likewise did not reveal associations with PD [14]. Future investigations should include screening of potential promoter as well as enhancer/silencer regions of the gene to finally exclude any lack of influence of *PINK1 *variation on PD manifestation. Yet, functional investigations of the *PINK1 *protein are necessary to identify potential interaction partners as candidates for additional mutation screening.

## Conclusion

Investigations of other genes involved in the mitochondrial pathway of the *PINK1 *gene are necessary to evaluate the exact role of mitochondrial impairment for common forms of PD.

## Materials and methods

### Patients and controls

The Norwegian cohort (n = 90) consists of patients suffering from late-onset PD (median age of onset 64.4 years, range from 49 to 78 years, standard deviation 7.9) originated from the Stavanger area of Western Norway. This population is known to be genetically quite homogeneous [11] and has previously been described in several clinical PD studies [15,16]. 16.7% of the patients presented a positive family history for PD concerning first degree relatives (siblings or parents). All patients meet the criteria for PD [15,17] and were thoroughly clinically examined. An ethnically matched control group of healthy blood donors was recruited in Bergen, Norway. The German cohort (n = 85) consists of patients of the Ruhr area suffering from late-onset PD (median age of onset 58.7 years, range from 45 to 79 years, standard deviation 8.7) diagnosed according to the UK Brain Bank criteria [18]. 16.5% of the patients presented a positive family history for PD concerning first degree relatives (siblings or parents). Ethnically matched control samples from senior healthy blood donors (median age 57.2 years, range from 42 to 68 years, standard deviation 5.7) were recruited at the neighbouring University Hospital of Essen (Germany). Population stratification was excluded for the controls by multiple microsatellites analyses. After receiving informed consent from the patients, peripheral blood samples were taken and genomic DNA was extracted following standard protocols. German (Bochum and Düsseldorf) and Norwegian (Bergen) ethics committees approved this study.

### SSCP, DHPLC, sequencing

The 8 coding exons of the *PINK1 *gene were amplified by polymerase chain reaction (PCR) in all patients using designed primer pairs adapted to the SSCP technique (Table 3). SSCP analysis according to standard procedure [19] was used to identify mutations and SNPs. In order to optimize mutation screening by SSCP analyses, PCR products were digested with different restriction enzymes depending on the lengths of their fragments [19] and screened in two different conditions. Selected samples with band shifts evidenced in SSCP analyses were confirmed by direct sequencing. The sequence reactions were run on an automated DNA sequencer (Applied Biosystems 377 XL, Foster City, USA) and analyzed with the ABI Prism™ 377 XL collection and convenient sequencing analysis software. SNP frequencies of the Q115L variation in patients and controls were determinated by using DHPLC analyses (WAVE^® ^system, Cheshire, UK, using software Wavemaker 4.1) according to established procedures.

**Table 3:** Primers for *PINK1 *gene analysis

Exon	Primer sequence	Product size (bp)
Ex 1	F 5'-AAGTTTGTTGTGACCGGCG-3' R 5'-CTTAGCTCCGTCCTCCGCT-3'	507
Ex 2	F 5'-CCTTCCTAGGCTCCCTGGC-3' R 5'-AAGATGGGCATTTTGAGAACATCT-3'	387
Ex 3	F 5'-GCTTACAAGGAACTTACCATTCTGC-3' R 5'-GTGCTGAGGACATAAGTGATGGAT-3'	240
Ex 4	F 5'-GATGTATCAGCTCCAGGCCCT-3' R 5'-TATTCTTTCCAGGTGTTGTATCTGATG-3'	286
Ex 5	F 5'-AAACGTATTGGGAGTCGTCGA-3' R 5'-CTCTAGTGCCCCTGGAGAGCT-3'	266
Ex 6	F 5'-CGAGTCTCCTGCATTCAGTGG-3' R 5'-GACATAGCAGGGCCTCTCAGAG-3'	265
Ex 7	F 5'-TCAGGTGATGTGCAGGACATG-3' R 5'-CAGAGGTTTCTACCCACACCG-3'	358
Ex 8	F 5'-GGACCAGAGAAGGGAAGACCC-3' R 5'-TCACGACACAGAGGATGCCA-3'	410

### Statistical analyses

SNP frequencies were compared by the χ^2 ^test. We considered P-values < 0.05 as significant.

## Authors' contributions

AMS carried out the molecular genetic studies, performed the statistical analysis and drafted the manuscript. MK participated in devising the study based on thoroughly clinical analysis of the patients. JPL supervised data collection and diagnosis of the Norwegian cohort. DW and TM provided the samples and performed clinical diagnostics of the German patient group. JTE conceived of the study, and participated in its design and coordination and helped to draft the manuscript. GD supervised AMS, especially the molecular studies. All authors read and approved the final manuscript.
